# Functional Role of AveC Residues Ser138 and Ala139 for Avermectin and Doramectin Biosynthesis in *Streptomyces avermitilis*

**DOI:** 10.3390/metabo16060409

**Published:** 2026-06-12

**Authors:** Zhangqun Li, Ling Zhang, Xiaofang Li, Mingjie Li, Haiyang Xia

**Affiliations:** 1 Institute of Biopharmaceuticals, School of Pharmaceutical Sciences, Taizhou University, Taizhou 318000, China; lizhangqun@126.com (Z.L.);; 2 Taizhou Key Laboratory of Pharmaceutical Biosynthesis, Taizhou 318000, China; 3 Shanghai Institute for Biomedical and Pharmaceutical Technologies (SIBPT), 2140 Xietu Road, Xuhui District, Shanghai 200032, China

**Keywords:** AveC, avermectin, doramectin, *Streptomyces avermitilis*

## Abstract

**Background:** Doramectin (CHC-B1) is an excellent antiparasitic drug produced by feeding cyclohexanecarboxylic acid (CHC) to *Streptomyces* *avermitilis bkd^−^* mutants. AveC, a bifunctional enzyme encoded by *aveC* (*sav_0940*), catalyzes the stereospecific spiroketalization and selective dehydration of dihydroxy ketone polyketide intermediates and modulates both the yield and the proportion of avermectin/doramectin in *Streptomyces avermitilis*. In our previous work, we constructed a strain harboring a synthetic aveC* gene encoding ten amino acid mutations, which produced nearly pure doramectin. However, the doramectin yield achieved only approximately 60% of the total doramectin and CHC-B2 output observed in the parental strain. **Methods:** To investigate the roles of Ser138 and Ala139 of AveC in the biosynthesis of doramectin and avermectin, site-directed mutagenesis was performed at both sites. The production and proportion of avermectin and doramectin were determined using high-performance liquid chromatography (HPLC). AlphaFold2-based molecular docking simulations were used to interpret the results. **Results:** Among the tested mutants, S138G, S138T, and A139H exhibited the highest doramectin production, achieving 143.87%, 151.22%, and 153.36% of the control level, respectively. Unfortunately, almost none of the tested mutants showed a positive effect on avermectin production. Molecular docking simulations revealed distinct affinities of these mutants for the dihydroxy ketone polyketide intermediate, both with and without a cyclohexyl group. Notably, all three mutants displayed larger substrate-binding cavity volumes compared with the wild-type enzyme, which likely facilitates doramectin synthesis by effectively accommodating the cyclohexyl moiety. Docking results further indicated that Ser138 and Ala139 are positioned within the binding cavity but probably do not directly participate in the dehydration activity. **Conclusions:** These findings suggest that optimizing cavity size through residue substitutions can enhance substrate specificity for doramectin production while preserving catalytic functionality.

## 1. Introduction

Avermectin (AVM), a hexadecyl macrolide produced by *Streptomyces avermitilis*, is one of the most widely used biopesticides in the world. The wild-type strain of *S. avermitilis* generates eight AVM components, including four major compounds (A1a, A2a, B1a, and B2a) and four minor compounds (A1b, A2b, B1b, and B2b). Among these, B1a exhibits the highest insecticidal activity and lowest toxicity [1]. Structural differences among AVM components arise from: (1) C5 methoxylation (A components) or hydroxylation (B components); (2) the presence of a C22–C23 double bond (1 components) or its hydrated product at C23 (2 components); and (3) C25 substitution with methylpropyl (a components) or isopropyl (b components) (Figure S1) [2].

The derivatives of AVM, including ivermectin, eprinomectin and doramectin, play critical roles in agriculture, veterinary medicine, and human healthcare. Doramectin (Figure S1), the third-generation AVM derivative, is renowned for its efficacy against both endoparasites and ectoparasites, offering a broader spectrum and higher potency than native AVM. It is produced in the *bkd^−^* (branched-chain-2-oxo acid dehydrogenase encoding gene) and *aveD* (5-O-methyltransferase encoding gene) mutants of *S. avermitilis* cultivated with cyclohexanecarboxylic acid (CHC) as a precursor [3,4,5,6].

**Figure 1:**
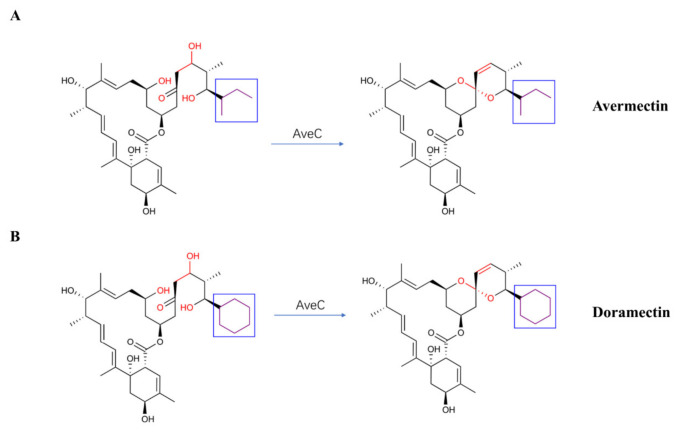
Function of AveC in the conversion of avermectin and doramectin. AveC catalyzes stereospecific spiroketalization and optional dehydration of a dihydroxy ketone polyketide intermediate to determine the ratio of component 1 to component 2 in AVM (**A**) and doramectin (**B**) biosynthesis.

The 65 kb AVM biosynthetic gene cluster (BGC) was cloned and characterized in 1999, and the *S. avermitilis* genome was sequenced in 2001, significantly advancing research on this organism [7,8]. The BGC encodes four polyketide synthases (AveA1–AveA4), seven enzymes for deoxysugar biosynthesis (AveBII–AveBVIII), five tailoring enzymes (AveC, AveD, AveE, AveF, and AveBI) and a pathway-specific regulator (AveR) (Figure S2) [8]. AveC, encoded by a gene located in the *aveA1–aveA2–aveC* operon, catalyzes stereospecific spiroketalization and optional dehydration of a dihydroxy ketone polyketide intermediate, directly determining the ratio of component 1 to component 2 in AVM and its derivatives’ biosynthesis (Figure 1) [2,8,9,10,11].

Improved *aveC* variants were identified by semi-synthetic DNA shuffling [12]. A shuffled *aveC* encoding ten amino acid substitutions (D48E, A61T, R71L, A89T, L136P, T149S, F176C, G179S, V196A, E238D) achieved a CHC-B2:doramectin ratio of 0.07:1, a 23-fold improvement over the wild-type spirocyclase [12]. Studies by Stutzman-Engwall revealed that *aveC* mutations significantly impact doramectin production by altering the balance between spiroketal formation (linked to yield) and optional dehydration (critical for component ratio) [13]. Notably, the Ser138 and Ala139 residues of AveC were identified as key sites for its dual activity. Mutants S138T and A139T further improved the CHC-B2:CHC-B1 ratio to 0.84:1 and increased total production (56.8 mg/L CHC-B1 + CHC-B2) [13].

The tertiary structure and activity of a protein are determined by the physicochemical properties of its constituent amino acids. The 20 proteinogenic amino acids can be grouped into three major classes, namely, hydrophobic, hydrophilic, and charged, based on their physicochemical properties [14]. To explore the relationship among amino acid properties, AveC activity and natural product yields, Ser138 and Ala139 were mutated to determine the yields of doramectin and AVM. Furthermore, the same AveC mutations were analyzed in both doramectin and AVM biosynthesis to explore the differences in AveC function between these two pathways, which remain incompletely understood.

AlphaFold (AlphaFold2) is an AI system developed by Google DeepMind that predicts a protein’s 3D structure from its amino acid sequence. It regularly achieves accuracy competitive with experiment [15]. Molecular docking is a computational technique used to predict substrate specificity by spatial and energy matching [16]. It uses computer-assisted methods to simulate the interaction between enzymes and their substrates or other small molecules [17]. Docking plays a critical role in structure-based protein design and key residue verification. The combination of AlphaFold and molecular docking provides a new approach to investigate a broader range of enzyme-substrate interactions [18].

In this study, we investigated the functional roles of Ser138 and Ala139 in AveC through site-directed mutagenesis based on amino acids properties and propose a mechanistic model for how these residues influence substrate binding and catalytic specificity during doramectin and AVM biosynthesis by leveraging 3D structure prediction (AlphaFold2) and molecular docking analysis.

## 2. Materials and Methods

### 2.1. Strains, Plasmids, Primers, and Growth Conditions

The strains and plasmids used in this study are listed in Table S1, and the primers are listed in Table S2. *Escherichia coli* DH5α and ET12567/pUZ8002 were used for routine cloning and propagation of non-methylated DNA for conjugation into *S. avermitilis* strains, respectively [19]. *E. coli* was cultivated at 37 °C in Luria–Bertani (LB) broth or on agar supplemented with the appropriate antibiotics. *S. avermitilis* was grown on MS medium (2% mannitol, 2% soybean flour, 2% agar) at 28 °C for 5 to 7 days to induce sporulation. The fermentation medium for producing avermectin and doramectin by *S. avermitilis* was as described in Dang et al. [4]. Seed cultures and fermentation were performed at 28 °C and 220 rpm.

### 2.2. Genetic Manipulation

The vectors for conjugation were constructed through routine cloning. The protocols for genetic manipulation in *Streptomyces* were described by Kieser et al. [20]. The process of conjugation is briefly described as follows. A selected colony of *E. coli* ET12567/pUZ8002 was inoculated into 5 mL LB containing 50 µg/mL kanamycin, 25 µg/mL chloramphenicol and 50 µg/mL apramycin, and grown overnight at 37 °C. Then, 100 µL of the overnight culture was inoculated into 10 mL fresh LB with antibiotics as mentioned above and grown for another 4 h at 37 °C until the OD600 reached 0.4. The *E. coli* cells were washed twice with an equal volume of LB without antibiotics, and then resuspended in 1 mL LB. During washing of the *E. coli* cells, for each conjugation, 10 µL (10^8^) of *S. avermitilis* spores were added to 500 µL 2 × YT broth. The spores were subjected to heat shock at 50 °C for 10 min and then cooled to room temperature. Then, 0.5 mL of *E. coli* cell suspension was mixed with 0.5 mL heat-shocked spores, centrifuged briefly, and the pellet was resuspended in 200 µL of the remaining liquid. The cells were plated on MS agar with 10 mM MgCl_2_ and incubated at 30 °C for 16 to 20 h. The plate was overlaid with 1 mL of water that contains 0.5 mg nalidixic acid and 1.25 mg apramycin. Incubation was continued at 30 °C for 5 to 7 days to obtain colonies. The isolated colonies were identified by PCR and cryopreserved in 25% glycerol solution at −80 °C to ensure their stability.

### 2.3. Production and Analysis of Doramectin and Avermectin

The strains preserved in glycerol solution at −80 °C were inoculated onto the MS plates and incubated at 30 °C for 7 days. Then, 1 cm^2^ of strain culture on MS plates was taken and inoculated into 30 mL of VM broth (2% corn starch, 0.5% glucose, 1% soybean flour, 1% cotton seed flour, pH 7.3) in 250 mL flasks and cultured at 28 °C, 220 rpm. Then, 2.5 mL of 2-day-old cultures were transferred into 25 mL of FM broth (12% corn starch, 0.03% thermostable amylase, 1% soybean flour, 1% cotton seed flour, 0.5% yeast extract, 0.2% K_2_HPO_4_, 0.25% ZnSO_4_, 1% CoCl_2_, 0.1% NaCl, 0.3% Mg_3_(PO_4_)_2_, 0.7% CaCO_3_, pH 6.8–7.2) in 250 mL flasks and cultured at 28 °C, 220 rpm. For doramectin production, 100 µL of CHC (20% *w/v*) was added to each flask after 2 days, and the cultures were incubated for 12 days. For avermectin production, the culture time was 10 days. A total of 1 mL of fermentation broth was mixed with 4 mL methanol, vortexed and allowed to stand for 30 min, and then centrifuged at 4000× *g* for 10 min. The resulting liquid phase was filtered through a 0.22 µm membrane filter and analyzed using an Agilent 1260 Infinity II HPLC system equipped with a GL Sciences Inertsil column (ODS-3 5 µm, 4.6 × 150 mm). The column was eluted at a flow rate of 1 mL/min for 15 min with acetonitrile:methanol:H_2_O (45:45:10). Metabolites were monitored at a wavelength of 245 nm. Production was calibrated using standard doramectin (MeilunBio, MB5036-1, Dalian, China) and avermectin (provided by Hisun Pharmaceutical Inc., Taizhou, China). All experiments were conducted in at least triplicate, and representative results are shown. Data are shown as mean values ± standard deviation (SD) with scatter points. Statistical analysis was performed using two-tailed unpaired Student’s *t*-test (* *p* < 0.05, ** *p* < 0.01).

### 2.4. Structure Prediction and Molecular Docking Analysis

The 3D structures of AveC and its mutants were generated using AlphaFold2 on a local server with the ‘monomer_ptm’ models, and the structural models were analyzed using PyMOL version 2.5.1 (https://pymol.org/) [15]. The 3D structures were further verified by ERRAT, WHATCHECK, and PROCHECK on SAVESv6.1 (https://saves.mbi.ucla.edu/). The PDB files of the proteins were obtained and saved for further docking analysis.

The object ligand (SDF file) for the biosynthesis of doramectin and avermectin (Figure 1) was generated using Chem3D version 21.0, and docking was performed using AutoDock Vina [21]. The conformational energies of the receptors were minimized using the YASARA Energy Minimization server (https://www.yasara.org). The optimized docking models of protein-ligand complexes were identified by a comprehensive evaluation of binding structure, binding energy, and possible interactions between the ligand and key residues of the protein. The cavity volumes of AveC and its mutants were calculated using CavityPlus 2022 [22]. 2D and 3D diagrams of protein–ligand complexes were generated using MOE version 2019.0102 (https://www.chemcomp.com/moe/help/2019/index_tut.html, accessed on 1 May 2026).

## 3. Results

### 3.1. Effects of Ser138 and Ala139 Mutations in AveC on Doramectin Production in S. avermitilis

To investigate the impact of amino acid substitutions at Ser138 and Ala139 in AveC on doramectin biosynthesis, the high-yielding strain *S. avermitilis* DM209 (a derivative of the wild-type strain with deletions in *pks3*, *olm*, and *pte*) was used as the parental strain in this study [4]. Site-directed mutants of *aveC* were constructed using the integrative plasmid pSET152, driven by the constitutive strong promoter *erm*Ep*, and introduced into DM209 via conjugation. A control strain carrying the wild-type *aveC* under the same promoter was used for comparison. A strain containing the empty vector pSET152 was used as a blank control.

**Figure 2:**
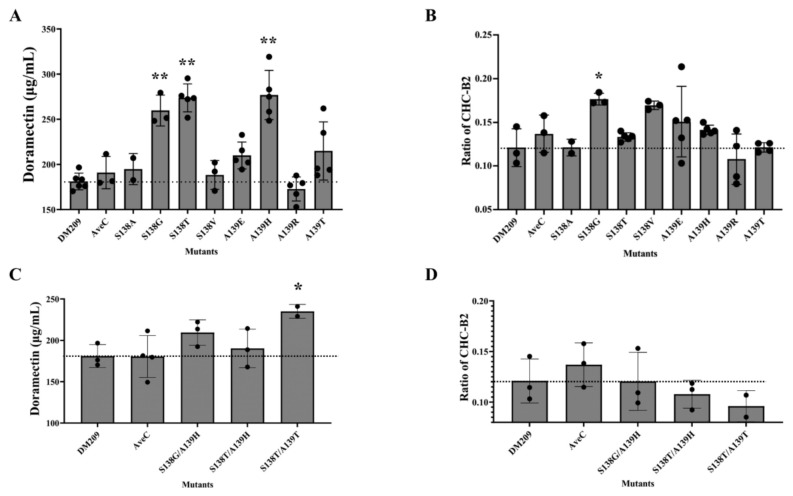
Ser138 and Ala139 mutations affect doramectin production in *S. avermitilis* DM209. (**A**) Single mutations of Ser138 and Ala139 affect the production of doramectin in *S. avermitilis*. (**B**) Single mutations of Ser138 and Ala139 affect the ratio of CHC-B2:doramectin in *S. avermitilis*. (**C**) Combination mutations of Ser138 and Ala139 affect the production of doramectin in *S. avermitilis*. (**D**) Combination mutations of Ser138 and Ala139 affect the ratio of CHC-B2:doramectin in *S. avermitilis*. Samples of 12-day fermentation broth from single mutants of Ser138 (DM S138A, DM S138G, DM S138T, DM S138V) and Ala139 (DM A139E, DM A139H, DM A139R, DM A139T), as well as from combination mutants (DM S138G/A139H, DM S138T/A139H, DM S138T/A139T), were used to analyze doramectin yield and the ratio of CHC-B2:doramectin in *S. avermitilis*. Strain DM209 in this figure is the blank control with the empty vector pSET152. Strain AveC is the control with the wild-type *aveC* under the same promoter. Data are shown as mean values ± SD with scatter points. Statistical analysis was performed using two-tailed unpaired Student’s *t*-test (* *p* < 0.05, ** *p* < 0.01). All experiments were performed in at least triplicate and representative results are shown.

The fermentation results showed that for residue Ser138, mutants with conservative amino acid substitutions such as S138G and S138T exhibited significantly enhanced doramectin yields when compared to the control strain (143.87% and 151.22% of the control, corresponding to 259.68 and 273.74 μg/mL). However, no significant improvements were observed in non-conservative mutants S138A and S138V (Figure 2A). At residue Ala139, the nature of the amino acid charge did not influence doramectin yield. A139H displayed a remarkable increase in doramectin production, reaching 153.36% of the control (276.80 μg/mL), while A139E, A139R and A139T showed no significant improvement (Figure 2A). An additional copy of wild-type *aveC* had a slight effect on doramectin production. To further investigate the effect of both sites on doramectin yield, combination mutants (S138G/A139H, S138T/A139H, and S138T/A139T) were evaluated. While these strains achieved doramectin levels of 116.02%, 105.42%, and 130.20% (209.41, 190.27, and 235.01 μg/mL, respectively) relative to the control, all combination mutants underperformed relative to the single mutants S138G, S138T, and A139H (Figure 2C). This suggests that double mutations may not bring synergistic enhancement of doramectin production.

Beyond total yield, the CHC-B2:doramectin ratio is a critical quality parameter for industrial applications. This ratio was calculated based on the integration area of HPLC peaks for CHC-B2 and doramectin. Notably, mutants A139R, S138T/A139H, and S138T/A139T exhibited reduced CHC-B2:doramectin ratios (0.11, 0.11, and 0.09, respectively) compared with the blank control DM209 (0.12) (Figure 2B,D). The S138T/A139T double mutant demonstrated the most significant improvement in minimizing CHC-B2 accumulation while maintaining high doramectin output (Figure 2D).

### 3.2. Effects of Ser138 and Ala139 Mutations in AveC on Avermectin Production in S. avermitilis

**Figure 3:**
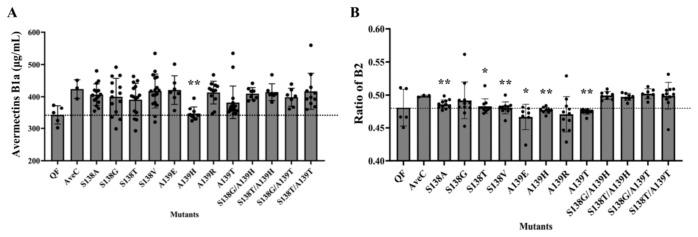
Ser138 and Ala139 mutations affect avermectin production in *S. avermitilis* QF-1. (**A**) Mutations of Ser138 and Ala139 affect the production of avermectin B1a in *S. avermitilis* QF-1. (**B**) Mutations of Ser138 and Ala139 alter the ratio of avermectin component 2 (B2) in *S. avermitilis* QF-1. Samples of 10-day fermentation broth from single mutants of Ser138 (QF S138A, QF S138G, QF S138T, QF S138V) and Ala139 (QF A139E, QF A139H, QF A139R, QF A139T), as well as from combination mutants (QF S138G/A139H, QF S138T/A139H, QF S138G/A139T, QF S138T/A139T), were used to analyze avermectin B1a yield and the B2 ratio in *S. avermitilis* QF-1. Strain QF in this figure is the blank control with the empty vector pSET152. Strain AveC is the control with the wild-type *aveC* under the same promoter. Data are shown as mean values ± SD with scatter points. Statistical analysis was performed using two-tailed unpaired Student’s *t*-test (* *p* < 0.05, ** *p* < 0.01). All experiments were performed in at least triplicate and representative results are shown.

The yields and ratios of doramectin demonstrated that mutations at Ser138 and Ala139 in AveC influence doramectin biosynthesis. Given that avermectin B1a (AVM-B1a) is the most biologically active component of the avermectin family, we extended this investigation to assess the impact of these mutations on AVM-B1a production in an industrial *S. avermitilis* strain QF-1. To evaluate the role of AveC mutations, the same batches of plasmids, including wild-type and mutant variants of *aveC*, were introduced into strain QF-1 via conjugation. The strain QF-1 containing pSET152 was used as the blank control. Notably, strains harboring an additional copy of wild-type *aveC* exhibited the highest AVM-B1a production among all tested mutants (Figure 3A). In contrast, most mutants—particularly those with dual-site mutations (e.g., S138G/A139T)—showed a shift toward avermectin B2 (AVM-B2) synthesis, indicating that *aveC* mutations differentially regulate the dehydration activity to produce AVM-B1a and AVM-B2 (Figure 3B).

Interestingly, mutant A139H even showed a negative effect on avermectin production. The production of avermectin B1a (Figure 3A) and AVMs (Figure S3) was similar to that of the original strain QF-1 carrying the empty vector pSET152. It appears that this copy of mutated AveC has no activity toward the dihydroxy ketone polyketide intermediate. However, as shown in Figure 2A, this mutation can promote doramectin production. This suggests that mutation A139H has stronger activity toward the dihydroxy ketone polyketide intermediate that bears a cyclohexyl group. These results demonstrate that AveC mutations regulate AVM biosynthesis in a context-dependent manner, with divergent impacts on doramectin and avermectin production.

### 3.3. Molecular Docking of AveC Mutants with Putative Substrates for Doramectin and Avermectin Biosynthesis

Due to the lack of structural information for AveC caused by challenges in heterologous expression, advances in AI-based technologies, such as AlphaFold2, have provided reliable strategies for predicting protein 3D structures. In this study, we employed AlphaFold2 to model the structure of AveC and its key mutants (S138G, S138T, A139H, A139T, S138T/A139H, S138T/A139T). Molecular docking simulations were performed using putative substrates proposed by Sun et al. to investigate their interactions with AveC variants [10].

**Figure 4:**
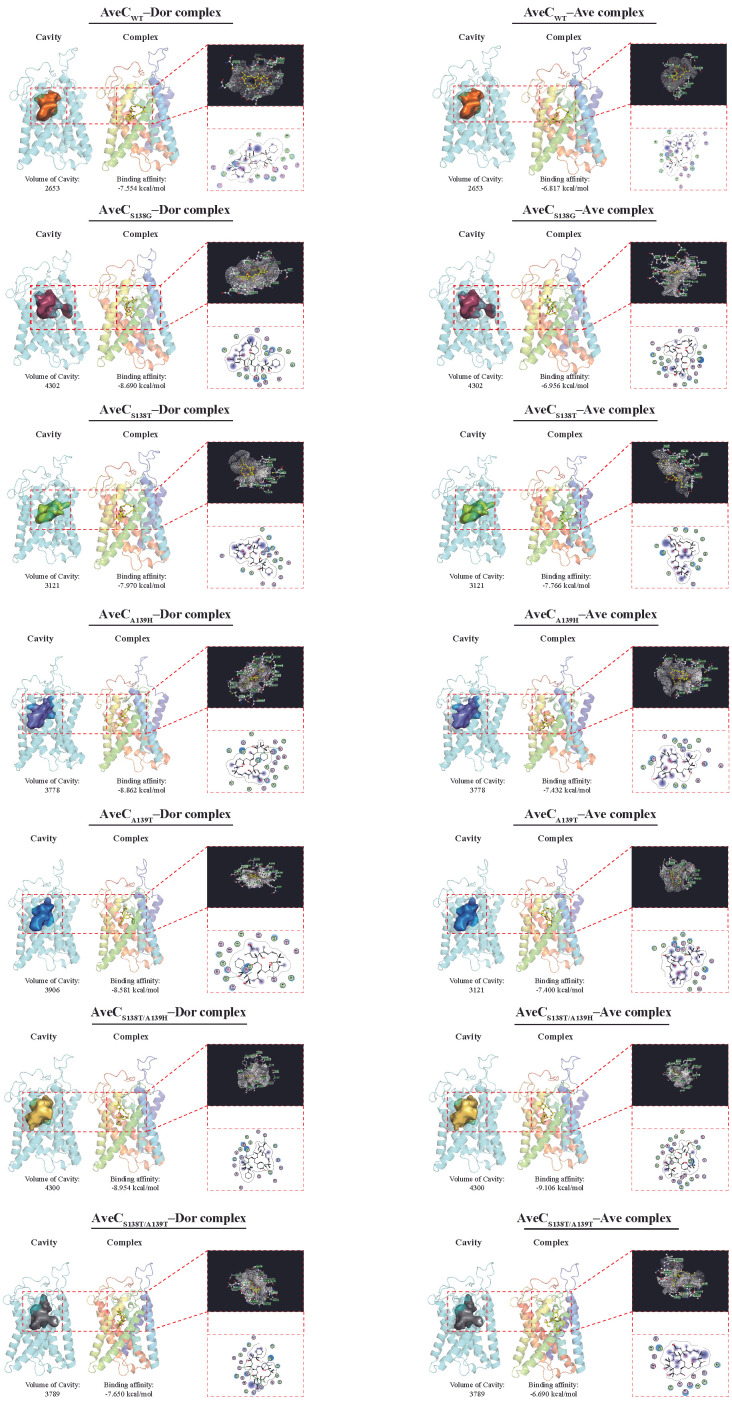
Evaluation of cavity volume and binding affinity of Ser138 and Ala139 mutants in AveC. Evaluation of cavity volume and binding affinity with doramectin- (referred to as Dor) and avermectin- (referred to as Ave) related substrates in Ser138 and Ala139 mutants in AveC by AlphaFold2.

Most mutants that enhanced doramectin production exhibited increased affinity for the proposed doramectin-related substrates (dihydroxy ketone polyketide intermediate with a cyclohexyl group) (Figure 4, Table 1). Structural analysis revealed that these mutants formed larger substrate-binding cavities compared with the wild-type enzyme, potentially accommodating substrates with a cyclohexyl group more effectively. This structural expansion likely explains the observed enhancement in doramectin biosynthesis by mutants S138G, S138T, A139H, A139T, and S138T/A139T.

Docking results indicated that Ser138 and Ala139 are not catalytic residues in the dehydration or spiroketalization reactions. Instead, these residues appear to modulate substrate specificity by influencing the size and geometry of the binding cavity. These two sites probably play an important role in determining substrate specificity. The absence of direct interactions between Ser138 and Ala139 and the substrates further supports their role in structural regulation rather than direct catalysis.

## 4. Discussion

Doramectin, a fermentation-derived veterinary drug used for controlling parasites in husbandry (e.g., gastrointestinal roundworms, lungworms, eyeworms, grubs, sucking lice, and mange mites), requires improved production efficiency to meet industrial demands [23,24,25,26]. Previous studies have identified AveC as a limiting factor in doramectin biosynthesis, making its optimization a critical target for strain improvement [10,12,13]. In this work, site-directed mutagenesis of *aveC* revealed that Ser138 and Ala139 are key residues influencing doramectin yield. Mutants S138G, S138T, and A139H significantly enhanced doramectin production but had minimal or negative effects on avermectin biosynthesis. The effects of changed amino acid properties on enzyme activity lacked a consistent pattern. Results of structural and docking analyses indicated that these mutants exhibit enhanced substrate affinity and improved accommodation of doramectin-related precursors due to expanded binding cavities. These findings suggest that larger binding cavities likely facilitate the biosynthesis of doramectin. However, the S138T/A139H double mutant has the highest predicted binding affinity to the avermectin precursor (−9.106 kcal/mol), but it does not show the highest AVM-B1a yield in the QF-1 strain (Figure 3A). We propose that the larger binding cavity of S138T/A139H was not suitable for the incorporation of avermectin substrates.

To assess the impact of *aveC* mutations on AVM production, we evaluated the same mutants in an industrial *S. avermitilis* strain. Most mutants showed no significant improvement in AVM yield, and the A139H mutant even reduced AVM production compared with the wild-type AveC. This suggests that the A139H mutation is deleterious to AVM biosynthesis. In contrast, Hao et al. engineered *aveC* to improve the B1a:B2a ratio in a high-yielding strain (A229) by introducing an eight-amino-acid mutation under the kasOp* promoter [9]. Their optimized *aveC8m* variant increased the B1a:B2a ratio from 0.99 to 1.33 and elevated total B1a + B1b production from 12,927 μg/mL (B1a: 6447 μg/mL; B1b: 6480 μg/mL) to 14,244 μg/mL (B1a: 8120 μg/mL; B1b: 6124 μg/mL) [9]. This multicopy overexpression strategy further underscores the rate-limiting role of AveC in AVM biosynthesis. In this study, combination mutants decreased the ratio of B1 in QF-1, which differs from that observed with single-site mutants.

Despite progress, structural characterization of AveC was limited due to challenges in heterologous expression, as reported by Sun et al. [10]. Our preliminary computational models provide insights into the functional role of AveC but await experimental validation. High-throughput saturation mutagenesis of *aveC* is necessary to systematically identify beneficial mutations. Large datasets of mutations and AveC production yields would be helpful for elucidating the catalytic mechanism of AveC through AI-assisted predictive modeling. These approaches could enable precise engineering of AveC for developing high-yielding strains for both doramectin and avermectin production in the future.

## 5. Conclusions

By site-directed mutagenesis of *aveC*, Ser138 and Ala139 were shown to be the two key residues for the biosynthesis of doramectin in *S. avermitilis*. It was proposed that the beneficial mutations of Ser138 and Ala139 likely form a larger cavity volume to facilitate the accommodation of doramectin intermediates with the cyclohexyl group.

## Figures and Tables

**Table 1:** Cavity volume and binding affinity of Ser138 and Ala139 mutants in AveC predicted by AlphaFold2. The cavity volume and binding affinity of Ser138 and Ala139 mutants in AveC for doramectin-related (referred to as Dor) and avermectin-related (referred to as Ave) substrates are predicted by AlphaFold2.

Strains	AveC-Dor Complex		AveC-Ave Complex	
	Volume of Cavity	Binding Affinity (kcal/mol)	Volume of Cavity	Binding Affinity (kcal/mol)
AveC_WT_	2653	−7.554	2653	−6.817
AveC_S138G_	4302	−8.690	4302	−6.956
AveC_S138T_	3121	−7.970	3121	−7.766
AveC_A139H_	3778	−8.862	3778	−7.432
AveC_A139T_	3906	−8.581	3121	−7.400
AveC_S138T/A139H_	4300	−8.954	4300	−9.106
AveC_S138T/A139T_	3789	−7.650	3789	−6.690

## Data Availability

The original contributions presented in this study are included in the article/Supplementary Materials. Further inquiries can be directed to the corresponding author.

## Supplementary Materials

The following supporting information can be downloaded at: https://www.mdpi.com/article/10.3390/metabo16060409/s1, Figure S1. The chemical structures of avermectin and doramectin. The structures of avermectin (A) and doramectin (B). Figure S2. The avermectin biosynthetic gene cluster in *S. avermitilis*. Figure S3. Effects of Ser138 and Ala139 mutations on avermectin production in *S. avermitilis* QF-1. Table S1. List of plasmids and strains used in this study. Table S2. List of primers used in this study. References [4,27,28] are cited in the supplementary materials.
