# Supplementary material for: Functional Role of AveC Residues Ser138 and Ala139 for Avermectin and Doramectin Biosynthesis in Streptomyces avermitilis
Source: Metabolites. 2026 Jun 12;16(6):409. doi: 10.3390/metabo16060409 (PMC13303154; doi:10.3390/metabo16060409)
Supplement: Supplementary file 1 [file metabolites-16-00409-s001.zip › Electronic Supplementary Materials.pdf]

Electronic Supplementary Material for

Functional Role of AveC Residues Ser138 and Ala139 for Avermectin and Doramectin Biosynthesis in *Streptomyces avermitilis*

Zhangqun Li[1,2], Ling Zhang[1,2], Xiaofang Li[1,2], Mingjie Li[3], Haiyang Xia[1,3,*]

1 Institute of Biopharmaceuticals, School of Pharmaceutical Sciences, Taizhou University, Taizhou 318000, China

2 Taizhou Key Laboratory of Pharmaceutical Biosynthesis, Taizhou 318000, China

3 Shanghai Institute for Biomedical and Pharmaceutical Technologies (SIBPT), Shanghai 200032, China

* Correspondence: hyxia@sibpt.cn

A

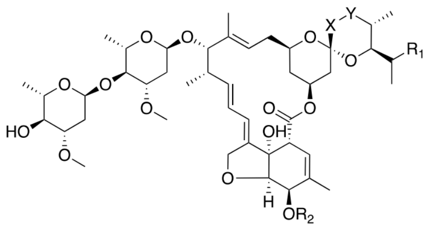

Avermectin Components	R_1	R_2	X-Y
A1a	C_2H_5	CH_3	CH=CH
A1b	CH_3	CH_3	CH=CH
A2a	C_2H_5	CH_3	CH_2-CH(OH)
A2b	CH_3	CH_3	CH_2-CH(OH)
B1a	C_2H_5	H	CH=CH
B1b	CH_3	H	CH=CH
B2a	C_2H_5	H	CH_2-CH(OH)
B2b	CH_3	H	CH_2-CH(OH)

B

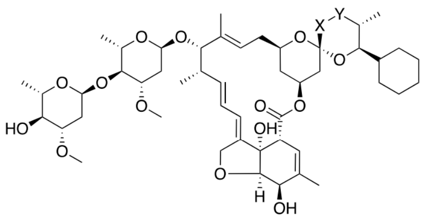

Components	X-Y
CHC-B1(doramectin)	CH=CH
CHC-B2	CH_2-CH(OH)

Figure S1. The chemical structures of avermectin and doramectin.

The structures of avermectin (A) and doramectin (B).

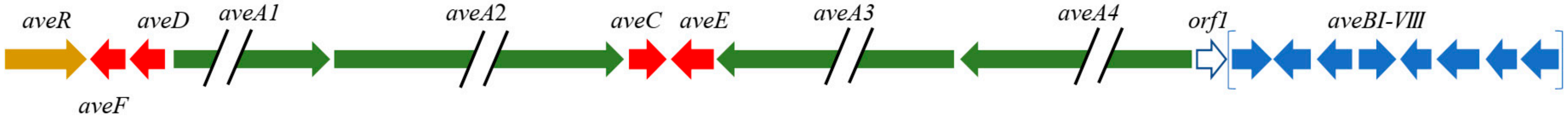

Figure S2. The avermectin biosynthetic gene cluster in *S. avermitilis.*

Schematic physical map of the avermectin biosynthetic gene cluster. Each arrow represents an open reading frame (ORF). Green, the polyketide synthase encoding genes (*aveA1~4*) responsible for polyketide skeleton assembly; blue, the genes involved in oleandrose biosynthesis and its transglycosylation to polyketide-derived aglycons; red, postpolyketide modification; yellow, the regulator gene; blank, the gene with unknown function.

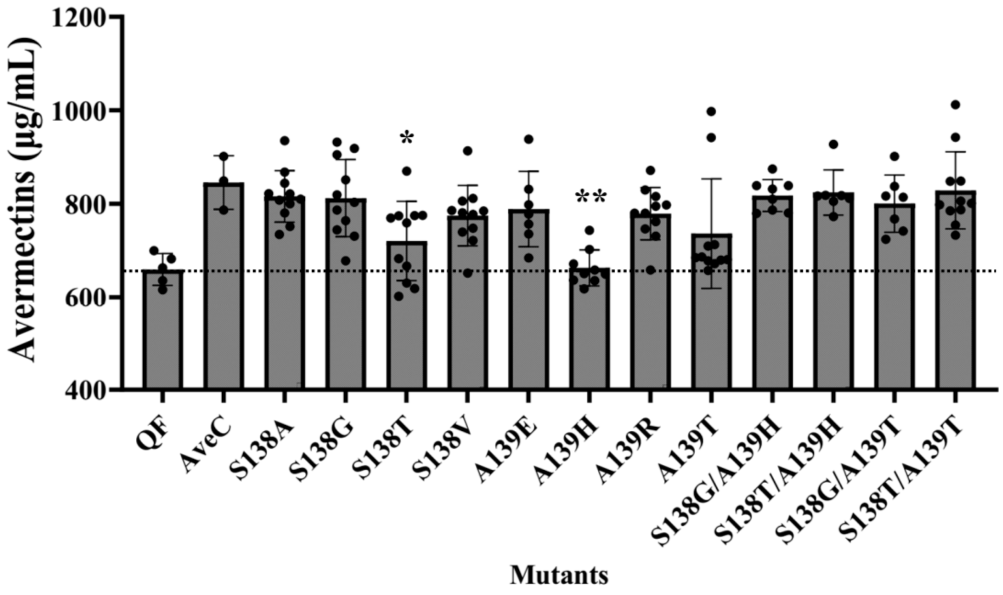

Figure S3. Effects of Ser138 and Ala139 mutations on avermectin production in *S. avermitilis* QF-1.

The avermectin yields in Ser138 and Ala139 mutants of AveC. Samples of 10-day fermentation broth from single mutants of Ser138 (QF S138A, QF S138G, QF S138T, QF S138V) and Ala139 (QF A139E, QF A139H, QF A139R, QF A139T), as well as from combination mutants (QF S138G/A139H, QF S138T/A139H, QF S138G/A139T, QF S138T/A139T), were used to analyze avermectin production in *S. avermitilis* QF-1. Strain QF in this figure is the blank control with the empty vector pSET152. Strain AveC is the control with the wild-type *aveC* under the same promoter. Data are shown as mean values ± SD with scatter points. Statistical analysis was performed using two-tailed unpaired Student's *t*-test (*p $<$0.05, **p$<$0.01). All experiments were performed in at least triplicate, and representative results are shown.

Table

Table S1. List of plasmids and strains used in this study

Plasmids/Strains	Descriptions	Source or references
**Plasmids**		
pSET152	pUC19 *ori*, *ΦC31 int/attP*, *aac(3)IV*, *lacZα*, and *oriT* RK2	[27]
pHX55	pUC19 ori, *bla*, *ermEp**	[28]
pSET152-aveC	pSET152 carrying *aveC* under the control of *ermEp**	This study
pSET152-S138A	pSET152 carrying Ser138 mutation to Ala138 of *aveC* under the control of *ermEp**	This study
pSET152-S138G	pSET152 carrying Ser138 mutation to Gly138 of *aveC* under the control of *ermEp**	This study
pSET152-S138T	pSET152 carrying Ser138 mutation to Thr138 of *aveC* under the control of *ermEp**	This study
pSET152-S138V	pSET152 carrying Ser138 mutation to Val138 of *aveC* under the control of *ermEp**	This study
pSET152-A139E	pSET152 carrying Ala139 mutation to Glu139 of *aveC* under the control of *ermEp**	This study
pSET152-A139H	pSET152 carrying Ala139 mutation to His139 of *aveC* under the control of *ermEp**	This study
pSET152-A139R	pSET152 carrying Ala139 mutation to Arg139 of *aveC* under the control of *ermEp**	This study
pSET152-A139T	pSET152 carrying Ala139 mutation to Thr139 of *aveC* under the control of *ermEp**	This study
pSET152-S138T/A139T	pSET152 carrying S138 Ala139 mutations to Thr138 Thr139 of *aveC* under the control of *ermEp**	This study
pSET152-S138G/A139T	pSET152 carrying S138 Ala139 mutations to Gly138 Thr139 of *aveC* under the control of *ermEp**	This study

pSET152-S138G/A139H	pSET152 carrying S138 Ala139 mutations to Gly138 His139 of *aveC* under the control of *ermEp**	This study
pSET152-S138T/A139H	pSET152 carrying S138 Ala139 mutations to Thr138 His139 of *aveC* under the control of *ermEp**	This study
**Strains**		
E.coli		
DH5α	Strain used to amplify target plasmids	TaKaRa
ET12567/pUZ8002	A methylation-deficient *E. coli* with helper plasmid pUZ8002 as donor strain for conjugation	[4]
Streptomyces avermitilis		
DM209	Δ*pks3*-Δ*olm*-Δ*pte* mutant of doramectin producing strain	[4]
QF-1	A avermectins producing strain derived from strain MMR630	This study
DM152	DM209 carrying pSET152	This study
QF152	QF-1 carrying pSET152	This study
DM*aveC*	DM209 carrying pSET152-*aveC*	This study
QF*aveC*	QF-1 carrying pSET152-*aveC*	This study
DM S138A	DM209 carrying pSET152-S138A	This study
DM S138G	DM209 carrying pSET152-S138G	This study
DM S138T	DM209 carrying pSET152-S138T	This study
DM S138V	DM209 carrying pSET152-S138V	This study
DM A139E	DM209 carrying pSET152-A139E	This study
DM A139H	DM209 carrying pSET152-A139H	This study
DM A139R	DM209 carrying pSET152-A139R	This study
DM A139T	DM209 carrying pSET152-A139T	This study
DM S138T/A139T	DM209 carrying pSET152-S138T/A139T	This study
DM S138G/A139T	DM209 carrying pSET152-S138G/A139T	This study
DM S138G/A139H	DM209 carrying pSET152-S138G/A139H	This study

DM S138T/A139H	DM209 carrying pSET152-S138T/A139H	This study
QF S138A	QF-1 carrying pSET152-S138A	This study
QF S138G	QF-1 carrying pSET152-S138G	This study
QF S138T	QF-1 carrying pSET152-S138T	This study
QF S138V	QF-1 carrying pSET152-S138V	This study
QF A139E	QF-1 carrying pSET152-S139E	This study
QF A139H	QF-1 carrying pSET152-S139H	This study
QF A139R	QF-1 carrying pSET152-S139R	This study
QF A139T	QF-1 carrying pSET152-S139T	This study
QF S138T/A139T	QF-1 carrying pSET152- S138T/A139T	This study
QF S138G/A139T	QF-1 carrying pSET152- S138G/A139T	This study
QF S138G/A139H	QF-1 carrying pSET152- S138G/A139H	This study
QF S138T/A139H	QF-1 carrying pSET152- S138T/A139H	This study

Table S2. List of primers used in this study

Primers name	Sequences(5'→3')[a]	Comments
AveC-F	gcaggtcgactctaggtggtggtgtgggccggg	Amplify the *aveC* gene
AveC-R	ccgcggatcctctagtcagtaagcggccggcgc	Amplify the *aveC* gene
S138A-F	ggaaatgccgccggcgGCCgcctccgtctgcatg	Site-directed mutagenesis
S138A-R	catgcagacggaggcGGCcgccggcggcatttcc	Site-directed mutagenesis
S138G-F	ggaaatgccgccggcgGGCgcctccgtctgcatg	Site-directed mutagenesis
S138G-R	catgcagacggaggcGCCcgccggcggcatttcc	Site-directed mutagenesis
S138T-F	ggaaatgccgccggcgACCgcctccgtctgcatg	Site-directed mutagenesis
S138T-R	catgcagacggaggcGGTcgccggcggcatttcc	Site-directed mutagenesis
S138V-F	ggaaatgccgccggcgGTCgcctccgtctgcatg	Site-directed mutagenesis
S138V-R	catgcagacggaggcGACcgccggcggcatttcc	Site-directed mutagenesis
A139E-F	atgccgccggcgtcgGAGtccgtctgcatgtcg	Site-directed mutagenesis
A139E-R	cgacatgcagacggaCTCcgacgccggcggcat	Site-directed mutagenesis
A139H-F	atgccgccggcgtcgCACtccgtctgcatgtcg	Site-directed mutagenesis
A139H-R	cgacatgcagacggaGTGcgacgccggcggcat	Site-directed mutagenesis
A139R-F	atgccgccggcgtcgCGCtccgtctgcatgtcg	Site-directed mutagenesis
A139R-R	cgacatgcagacggaGCGcgacgccggcggcat	Site-directed mutagenesis
A139T-F	atgccgccggcgtcgACCtccgtctgcatgtcg	Site-directed mutagenesis
A139T-F	cgacatgcagacggaGGTcgacgccggcggcat	Site-directed mutagenesis
S138G/A139T-F	ggaaatgccgccggcgGGCACCtccgtctgcatgtcg	Site-directed mutagenesis
S138G/A139T-R	cgacatgcagacggaGGTGCCcgccggcggcatttcc	Site-directed mutagenesis
S138G/A139H-F	ggaaatgccgccggcgGGCCACtccgtctgcatgtcg	Site-directed mutagenesis
S138G/A139H-R	cgacatgcagacggaGTGGCCcgccggcggcatttcc	Site-directed mutagenesis
S138T/A139T-F	ggaaatgccgccggcgACCACCtccgtctgcatgtcg	Site-directed mutagenesis
S138T/A139T-R	cgacatgcagacggaGGTGGTcgccggcggcatttcc	Site-directed mutagenesis
S138T/A139H-F	ggaaatgccgccggcgACCCACtccgtctgcatgtcg	Site-directed mutagenesis
S138T/A139H-R	cgacatgcagacggaGTGGGTcgccggcggcatttcc	Site-directed mutagenesis
Apr-F	tgaccgactggaccttccttctga	PCR validation

Apr-R	cgaagatgggccacttggactgat	PCR validation

a：The capital letters represent the mutation sites.

Reference:

4. Dang, F.; Xu, Q.; Qin, Z.; Xia, H. Rationally improving doramectin production in industrial *Streptomyces avermitilis* strains. *Bioengineering (Basel)* **2023**, *10*.

27. Bierman, M.; Logan, R.; O'Brien, K.; Seno, E.T.; Rao, R.N.; Schoner, B.E. Plasmid cloning vectors for the conjugal transfer of DNA from *Escherichia coli* to *Streptomyces spp. Gene* **1992**, *116*, 43-49.

28. Li, X.; Yan, Y.; Xie, S.; Li, Z.; Xia, H. Enhancement of milbemycins production by phosphopantetheinyl transferase and regulatory pathway engineering in *Streptomyces bingchenggensis. World J Microbiol Biotechnol* **2023**, *39*, 278.
